# Improved Glycemia and Quality of Life Among Loop Users: Analysis of Real-world Data From a Single Center

**DOI:** 10.2196/40326

**Published:** 2022-10-24

**Authors:** Amy E Morrison, Kimberley Chong, Valerie Lai, Kate Farnsworth, Peter A Senior, Anna Lam

**Affiliations:** 1 Department of Endocrinology and Metabolism Department of Medicine University of Alberta Edmonton, AB Canada; 2 Diabetes Action Canada Toronto, ON Canada; 3 Alberta Diabetes Institute Edmonton, AB Canada

**Keywords:** type 1 diabetes, closed loop, automated insulin delivery, do-it-yourself

## Abstract

**Background:**

Despite do-it-yourself automated insulin delivery being an unapproved method of insulin delivery, an increasing number of people with type 1 diabetes (T1D) worldwide are choosing to use Loop, a do-it-yourself automated insulin delivery system.

**Objective:**

In this study, we aimed to assess glycemic outcomes, safety, and the perceived impact on quality of life (QOL) in a local Edmonton cohort of known Loop users.

**Methods:**

An observational study of adults with T1D who used Loop was performed. An assessment of glycemic and safety outcomes, HbA_1c_, time in range, hospital admissions, and time below range compared users most recent 6 months of Loop use, with their prior regulatory approved insulin delivery method. QOL outcomes were assessed using Insulin Dosing Systems: Perceptions, Ideas, Reflections, and Expectations, diabetes impact, and device satisfaction measures (with maximum scores of 100, 10, and 10, respectively) and semistructured interviews.

**Results:**

The 24 adults with T1D who took part in this study 16 (67%) were female, with a median age of 33 (IQR 28-45) years, median duration of diabetes of 22 (IQR 17-32) years, median pre-Loop HbA_1c_ of 7.9% (IQR 7.6%-8.3%), and a median duration of Loop use of 18 (IQR 12-25) months. During Loop use, the participants had median (IQR) values of 7.1% (6.5%-7.5%), 54 mmol (48-58) for HbA_1c_ and 76.5% (64.6%-81.9%) for time in range, which were a significant improvement from prior therapy (*P*=.001 and *P*=.005), with a nonsignificant reduction in time below range; 3.0 to 3.9 mmol/L (*P*=.17) and <3 mmol/L (*P*=.53). Overall, 2 episodes of diabetic ketoacidosis occurred in a total of 470 months of Loop use, and no severe hypoglycemia occurred. The positive impact of Loop use on QOL was explored in qualitative analysis and additionally demonstrated through a median Insulin Dosing Systems: Perceptions, Ideas, Reflections, and Expectations score of 86 (IQR 79-95), a median diabetes impact score of 2.8 (IQR 2.1-3.9), and a median device satisfaction score of 9 (IQR 8.2-9.4).

**Conclusions:**

This local cohort of people with T1D demonstrated a beneficial effect of Loop use on both glycemic control and QOL, with no safety concerns being highlighted.

## Introduction

### Background

Do-it-yourself (DIY) automated insulin delivery (AID) systems are user-designed systems that combine 2 regulated devices, an insulin pump that delivers a continuous subcutaneous insulin infusion and a continuous glucose monitor (CGM) that is controlled by an algorithm. Through this predictive algorithm, coded by the user, these systems facilitate an automated adjustment in insulin delivery, tailored to an individual’s requirements [1]. People with type 1 diabetes (T1D) are increasingly using these systems worldwide because the rapidly evolving software with extensive opportunities for customization helps individuals to achieve personalized glucose targets and reduce the burden of diabetes management [2].

DIY AID systems can be subclassified into system types (including AndroidAPS, FreeAPSX, and Loop) depending on the technology and the algorithms on which they run. These systems have not gained regulatory approval; users are effectively *hacking* licensed technology to run these algorithms and modulate their insulin delivery [3]. Recently, the first randomized controlled trial to highlight both the safety and the efficacy of AndroidAPS has been completed [4]. There are also multiple published studies, single-arm cohort studies, user self-reported pre-post data, and case series, reporting beneficial outcomes in glycemic control, quality of life (QOL), and reassuring safety data with DIY AID use. The studies have reported on individual system types or combinations of these [5-12].

Studies on DIY AID system use consistently report excellent glycemic outcomes, with very high time in range (TIR) and low time below range (TBR). These values far exceed those suggested as clinically recommended targets, achieved by only a minority of people with T1D [13]. Individuals choosing to use DIY AID are a select sample of people with T1D who are highly motivated to engage in self-care. Users are actively involved in optimizing glycemia with the aims of preventing diabetes-related morbidity, increasing life expectancy, and improving sleep quality [12].

Internet resources and social media platforms are currently the mainstay of guidance for DIY AID users [2]. These platforms have been used by enthusiasts in the field to collect outcome data [14]. The average ages of users receiving insulin delivery via a DIY system are reported to be 35.8 years (AndroidAPS), 33 years (OpenAPS), and 28.5 years (Loop), but the extensive benefits of these systems have been reported in studies of both adults and children, with 26% of users in a cross-sectional survey being aged <16 years [11]. Similar benefits have been observed across the 3 DIY AID system types, with the type of system studied usually being dependent on the geographical distribution of system users. Loop is the most commonly used DIY AID system in North America and AndroidAPS is the most commonly used DIY AID system in Europe [12]. To date, there have been no cohort studies performed in Canada to assess user outcomes for DIY AID users.

### Objectives

We sought to explore the experiences of adults using Loop at a single center in Canada. We aimed to assess quantitative outcomes in the form of glycemic, QOL, and safety data and also used a qualitative approach to gain a greater understanding of the lived experiences of Loop users.

## Methods

A cross-sectional study of current glycemia, experiences of Loop use, and QOL was performed in adults with T1D who were attending the Kaye Edmonton Clinic, which is part of the University of Alberta Hospital in Edmonton, Alberta, and were known to be currently using any form of DIY AID system.

### Ethics Approval

This study was approved by the University of Alberta Research Ethics Board (Study ID pro00111577).

### Participants

Prospective participants were identified and contacted by a member of their clinical team at the Kaye Edmonton Clinic. All participants were adults (aged ≥18 years) with T1D who were using a DIY AID system at the time of data collection. We arranged a semistructured interview with a member of the study team for participants after obtaining informed consent from them to take part in the study.

### Outcome Measures

Up to 6 months of most recent glucose data, while using Loop, were collected from the participants’ CGM download data, to record mean TIR 3.9 to 10.0 mmol/L (70-180 mg/dL), TBR 3.0 to 3.9 mmol/L (54-70 mg/dL), TBR; <3.0 mmol/L (<54 mg/dL), and time above range: >10.0 mmol/L (180 mg/dL). Where available, the same data were collected retrospectively from the participants’ glucose sensor data for the 6-month period before commencing Loop, while they were using their previous mode of insulin delivery. The participants’ laboratory HbA_1c_ readings (%) were collected from hospital records, including the most recent value with Loop use, in addition to the participant’s last reading before commencing Loop. The hospital records of all participants were reviewed for hospital admissions, specifically assessing the occurrence of severe hypoglycemia (SH) and diabetic ketoacidosis (DKA) throughout the duration of the participants’ Loop use. The University of Alberta Hospital uses an integrated medical record system, enabling data capture of admissions to any facility in the province.

**Figure 1:**
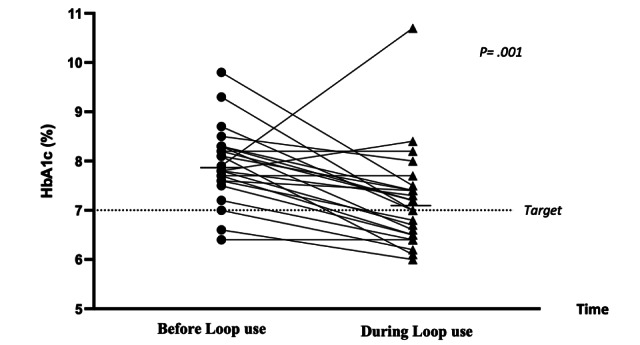
HbA_1c_ before Loop use and during 18 months of Loop use.

Semistructured interviews were arranged via telephone or through the use of the Zoom videoconferencing service (Zoom Video Communications, Inc) [15] between July and September 2021. A full interview transcript guide is available in Figure 1. Each interview was conducted by researchers AM and KC, with one asking questions while the other transcribed responses. During the interview process, demographic data were collected, including age, type of DIY AID system used, duration of DIY AID use, duration of diabetes, sex, ethnicity, occupation, and highest level of educational attainment. Participants were asked to report any episodes of SH that required the assistance of another person to treat and any occurrence of DKA during Loop use. Qualitative questions were related to participants’ reasons for commencing, challenges in its use, and support mechanisms with regard to using a DIY AID system as well as the benefits and barriers that they experienced with DIY AID use.

After the interviews, the participants electronically completed 2 validated questionnaires, Diabetes Impact and Device Satisfaction (DIDS) [16,17] and Insulin Dosing Systems: Perceptions Ideas Reflections and Expectations (INSPIRE) [18], evaluating their perceived impact of using DIY AID on their QOL. Full copies of these questionnaires are available in the appendices.

### Analysis

Descriptive statistical analysis and normality testing via the Shapiro-Wilk test were performed using GraphPad Prism (version 9.2.0 for macOS; GraphPad Software). A normal distribution was seen in both TIR and HbA_1c_ data before but not after Loop use, with additional skewed distributions being seen in age and QOL outcome measures. Therefore, data are reported as median (IQR) in the analysis of this cohort with nonparametric tests being used and statistical significance being defined as *P*<.05. Paired groups were compared using the Wilcoxon signed-rank test and unpaired data were compared using the Mann-Whitney *U* test, in addition to the correlation of variables using the Spearman correlation coefficient.

Qualitative interview data were coded deductively by the research team using NVivo 12 (QSR International) [19], after the data-driven inductive generation of the code structure (Multimedia Appendix 1). This deductive code structure was developed inductively from our data in addition to the consideration and inclusion of common themes identified in previous DIY AID user interview studies [20-22]. Overarching themes were constructed from the participants’ viewpoints and reflexive thematic analysis was performed by AM [23].

## Results

### Overview

A total of 24 adults with T1D participated in this cross-sectional study, with a median age of 33 (IQR 27.5-44.8) years and median duration of diabetes of 21.5 (IQR 17.3-32.0) years. All 24 participants were using the Loop subtype of DIY AID as their method of insulin delivery for a median duration of 18 (IQR 12-25) months, with a total of 470 months or 39.2 years of Loop use in the cohort. The demographic characteristics of this cohort of Loop users are described in Table 1. Of the 24 participants, the majority (n=16, 67%) were female and (n=22, 92%) White and over one-third (n=9, 38%) were employed in health care professions.

**Table 1:** Characteristics of study participants (N=24).

Characteristics		Values
Age (years), median (IQR)		33 (27.5-44.8)
Duration of diabetes (years), median (IQR)		21.5 (17.3-32.0)
Duration of Loop use (months), median (IQR)		18.0 (12.0-25.0)
**Sex, n (%)**		
	Male	8 (33)
	Female	16 (67)
**Ethnicity, n (%)**		
	White	22 (92)
	South Asian	1 (4)
	Mixed race	1 (4)
**Educational attainment, n (%)**		
	Master’s degree	4 (17)
	University degree	12 (50)
	Postsecondary certification or diploma	5 (21)
	High school	3 (13)
**Occupation, n (%)**		
	Health care professional	9 (38)
	Public servant	5 (21)
	Student	3 (13)
	Teacher	2 (8)
	Engineer	2 (8)
	Electrician	1 (4)
	Project manager	1 (4)
	Retired	1 (4)
**Glucose sensor use before Loop, n (%)**		
	Real-time CGM^a^	20 (83)
	Intermittently scanned CGM	3 (13)
	No sensor	1 (4)

^a^CGM: continuous glucose monitor.

### Glycemic Outcomes

**Figure 2:**
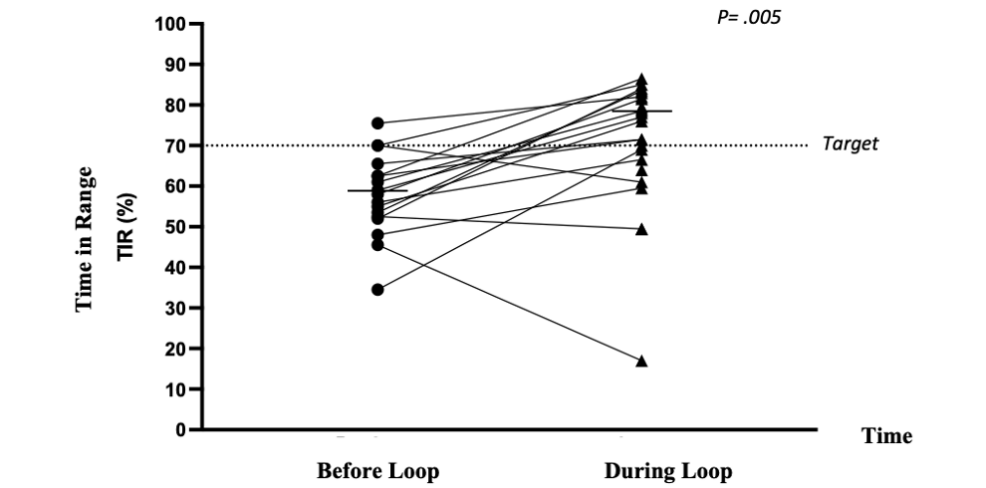
Time in range before Loop use and during Loop use.

HbA_1c_ values were available both before and after commencing Loop use for all participants. CGM data were available for 6 months before commencing Loop use for 71% (17/24) of the study participants, with a mean of 5.8 (SD 0.66) months of CGM data with Loop use being reviewed per participant. No significant differences in age, duration of diabetes, duration of Loop use, baseline HbA_1c_, or QOL outcome measure scores were seen between those participants with and without pre-Loop CGM data. Before Loop, median HbA_1c_ was 7.9% (IQR 7.6%-8.3%) or 63 (IQR 60-67) mmol/mol, and median TIR was 58% (IQR 52.3%-64.0%). A statistically significant improvement in these parameters was seen with Loop (*P*=.001 and *P*=.005). A median increase of 15% (IQR 6.3%-23.8%) in TIR was seen in 82% (20/24) of Loop users. Before Loop, 17% (4/24) of users achieved the clinical target of 70% TIR, in comparison with 67% (16/24) of users who achieved it with Loop use. HbA_1c_ reduction was seen in 79% (19/24) of users with Loop; the median rate of improvement was 0.8% (IQR 0.28%-1.18%). In addition, a significant reduction in time above range was demonstrated with the introduction of Loop (*P*=.008). Glycemic data are shown in Table 2 and Figures 1 and 2.

**Table 2:** Glycemic outcomes in users most recent 6 months of Loop use, in comparison with their prior insulin delivery method^a^.

Glycemic measure		Before Loop	After commencing Loop use	*P* value
Glycated hemoglobin (%), median (IQR)		8 (7.6-8.3)	7.1 (6.5-7.5)	.001
TIR^b^ (3.9-10 mmol/L, 70-180 mg/dL; %), median (IQR)		58.0 (52.3-64.0)	76.5 (64.6-81.9)	.005
**TBR^c^ (%), median (IQR)**				
	3.0-3.9 mmol/L, 54-70 mg/dL	1.5 (1.0-2.8)	1.3 (0.6-2.4)	.16
	<3.0 mmol/L, <54 mg/dL	0.5 (0.5-0.8)	0.5 (0.5-0.5)	.53
TAR^d^ (>10 mmol/L >180 mg/dL; %), median (IQR)		40.0 (31.5-46.5)	21.8 (15.4-33.25)	.008
Target HbA_1c_ (<7%), n (%)		2 (8.3)	10 (42)	—^e^
Target TIR (>70%), n (%)		3 (18)	16 (67)	—

^a^Data are median (IQR) and n (%). The Wilcoxon signed-rank test has been used to compare glycemic outcomes before Loop use and during the most recent 6 months of Loop use.

^b^TIR: time in range.

^c^TBR: time below range.

^d^TAR: time above range.

^e^Not available.

### Safety

Of the 24 participants, 2 (8%) experienced an episode of DKA, and no episodes of SH occurred in the cohort with Loop use. One episode of DKA was euglycemic and was associated with gastrointestinal infection and sodium-glucose cotransporter-2 inhibitor use; it required hospital admission, including intensive care unit stay for 4 days and was completely resolved. The other one was documented to be associated with a urinary tract infection; intensive care unit stay was not required and no insulin pump or Loop system failure was identified. These episodes of DKA occurred 15 and 11 months following starting Loop, respectively.

### QOL Measures

**Figure 3:**
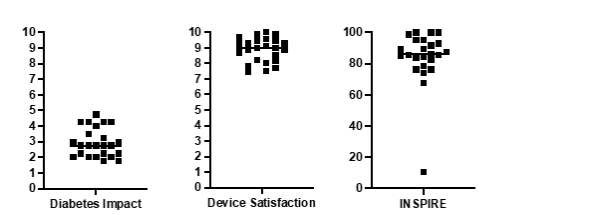
Quality of life outcome measures during Loop use. Scatter plots demonstrating diabetes impact out of 10 with lower scores being better, device satisfaction out of 10 with higher scores being better, and Insulin Dosing Systems: Perceptions, Ideas, Reflections, and Expectations scores out of 100 with higher scores being better. Median score line and individual values have been plotted. INSPIRE: Insulin Dosing Systems: Perceptions Ideas Reflections and Expectations.

The QOL measures collected following participant interviews by using the DIDS and INSPIRE questionnaires are shown in Figure 3 and Table 3. The median diabetes impact score was 2.8 (IQR 2.1-4.8) out of a maximum of 10, with lower scores indicating better outcomes. The median device satisfaction score was 9.0 (IQR 8.2-9.4) out of 10, with higher scores indicating better outcomes. The median INSPIRE score was 86.0 (79.5-94.6), with 100 being the maximum and optimal score. An examination of these QOL scores and of glycemic variables showed no significant positive correlations with TIR (*r*=0.024, *r*=0.007, and *r*=0.207; *P*=.41 or with HbA_1c_ (*r*=−0.163, *r*=−0.287, and *r*=−0.254; *P*=.38). A moderate correlation was seen between increased duration of Loop use and lower diabetes impact scores (*r*=−0.420, *P*=.04).

**Table 3:** Quality of life outcomes with automated insulin delivery system use; comparison of outcomes of Loop use in this cohort with outcomes of Tandem Control-IQ use in 2 other cohorts [16,24].

Quality of life measure	Outcome with Loop use, median (IQR)	Tandem Control-IQ 1, median (IQR) ^a^	Tandem Control-IQ 2, median (IQR)^b^
Diabetes impact score (maximum 10)	2.8 (2.1-4.8)	2.7 (1.8-3.7)	N/A^c^
Device Satisfaction score (maximum 10)	9.0 (8.2-9.4)	9.1 (8.4-9.8)	N/A
INSPIRE^d^ score (maximum 100)	86.0 (79.5-94.6)	N/A	87 (77.6-96.5)

^a^Two months of Tandem Control-IQ use [16].

^b^Six months of Tandem Control-IQ use [24].

^c^N/A: not applicable.

^d^INSPIRE: Insulin Dosing Systems: Perceptions Ideas Reflections and Expectations.

### Qualitative Interview Outcomes

#### Overview

The analysis of semistructured interview data highlighted frequent topics that participants had expressed as important in their lived experiences of Loop use. Overarching themes were constructed from these viewpoints, comprising empowerment and control, the daily impact of living with diabetes with Loop use, quantification of risk, and society’s understanding and awareness of Loop (Textbox 1).

**Thematic analysis outcomes with user experience examples.**

**Theme and user experience**

- Empowerment and control
  “The control I get; recognizing that I will go low and it stops the insulin. Loop provides more flexibility and personalization, and it’s more in my control, that’s why I would stick with a DIY over a Commercial system.” [31 years, female; 21 years type 1 diabetes (DM); 12 months of Loop use]
  “I feel a lot better all the time. My TIR is so much better. I have more freedom; I feel there is a little bit of that every time you get a device. Having Loop going on in the background to catch any mistakes is great. It makes me sleep better at night.” [24 years, female; 14.5 years DM; 5 months of Loop use]
  “I just feel that my control in my worst weeks with Loop are like my glucose levels in the best weeks when I was self-managing. I feel like Loop is like having a holiday from diabetes.” [29 years, female; 27 years DM; 7 months of Loop use]
  “Yes, just to note that the system has been so empowering. This disease can make you feel very powerless.” [49 years, female; 37 years DM; 7 months of Loop use]
- The daily impact of living with diabetes with Loop use
  “It has taken the hourly weight of diabetes off. Loop is the best thing that has entered my life.” [33 years, female; 22 years DM; 18 months of Loop use]
  “I have better control, reduced time worrying about diabetes but I would say I am spending more time managing my diabetes currently, as the system is relatively new to me.” [48 years, female; 35 years DM; 3 months of Loop use]
  “Using Loop there are more things to have to worry about, more tech to charge and make sure you have all the pieces with you when you go places, just more stuff to remember.” [31 years, female; 21 years DM; 12 months of Loop use]
  “Ordering the RileyLink took a while. Then, there was the time- building it, waiting. The financial aspect and finding the supplies. If you want to be on a Medtronic pump it is difficult to find one (522 or 722), or they are being sold for a very expensive price.” [27 years, female; 22 years DM; 40 months of Loop use]
- Quantification of risk
  “Yes, but I think there are risks to everything. There are risks to crossing the street, but that doesn’t mean you would never cross the street does it. As long as you take the time to figure out and correct your ratios and put all the correct information into the system, you definitely get out what you put in. You just really need to know your diabetes.” [24 years, female; 17 years DM; 39 months of Loop use]
  “Yes, it is safer than a regular pump- they remove the emotional element and decision-making and prevent snap decisions being made. The system is safe once the settings are correct, it is not safe with incorrect settings.” [51 years, male; 32 years DM; 17 months of Loop use]
  “There is a risk of the software being incorrect as the builders don't have the resources to test like big tech companies but at the same time anyone can review the algorithm so it is subject to a lot of scrutiny. I do worry what will happen if the developers move on to other projects.” [72 years, male; 19 years DM; 18 months of Loop use]
  “Yes, it is safe. The only thing that I sometimes think about is the issue that the Dexcom can have and how Loop only acts according to the information it gets from Dexcom. I have no actual issues with Looping itself.” [24 years, female; 14.5 years DM; 5 months of Loop use]
- Society’s understanding and awareness of Loop
  “I feel like it is just me and no one knows about it. Sometimes it can be a little bit lonely.” [22 years, female; 12 years DM; 12 months of Loop use]
  “The Looped Facebook group was the biggest thing. Loop docs website was very easy to follow. Support from diabetes team, I felt pretty lucky because there are other physicians that don’t approve of loop or help you with it, I know.” [27 years, female; 22 years DM; 40 months of Loop use]
  “I have had zero support since starting. I haven’t reached out to my DN and she may have been able to help, but pregnancy endos had no idea and were encouraging me to stop Looping even though I had found Loop very beneficial during pregnancy, especially in maintaining tight targets and avoiding severe hypoglycemia.” [29 years, female; 22 years DM; 25 months of Loop use]
  “My family were not supportive at first, they were not sure until they saw the a1c and how it worked. My care team’s lack of support also scared them, but now my family is very supportive. Also, my partner is very supportive, he would stay up to ensure it was working properly.” [24 years, female; 12 years DM; 15 months of Loop use]

#### Empowerment and Control

The principle of autonomy, with individual choice in selecting an optimal management regimen for their condition that was best suited to and most beneficial for them, was a prominently featured theme in why participants had chosen Loop. The feeling of dissatisfaction with a prior treatment option was described, with the need to make an individual choice to optimize their lifestyle:

Honestly in my work I felt like I needed the added security, something better than my pump. I had heard about Loop through social media and a diabetic influencer, I didn’t even know if I could do it in Canada, but enquired through the internet and then worked through the shared information on set up.

24 years, female; 17 years DM; 39 months of Loop use

Control was a term that participants frequently mentioned, referring to both this treatment choice component and glucose targets. Most of them included improvements in TIR and HbA_1c_ as motivating factors to commence and prominent benefits of Loop use. Increased lifestyle flexibility, particularly relating to diet and exercise patterns, was a commonly reported benefit:

I have more time and don’t have to worry as much about what I eat. I feel more flexible in eating schedules and working out. With Loop I can eat whenever I want and exercise when I want to, I can eat a surprise high carb meal for example.

24 years, female; 12 years DM; 15 months of Loop use

Another important benefit was the ability to sleep well overnight, being able to rely on Loop to ensure safety, particularly to avoid nocturnal hypoglycemia. Multiple participants reported struggling with nocturnal hypoglycemia before implementing Loop:

A year prior to looping I was having a lot of night-time lows and not waking up (didn’t feel them, didn’t hear CGM alerts), and would get phone calls from my mom.

27 years, female; 22 years DM; 40 months of Loop use

It was apparent that Loop offered peace of mind to both users and their friends and family members by preventing nocturnal hypoglycemia. Individuals who had struggled with this issue previously described the importance of this new aspect of control that Loop had enabled:

Having Loop going on in the background to catch any mistakes is great. It makes me sleep better at night.

24 years, female; 14.5 years DM; 5 months of Loop use

#### The Daily Impact of Living With Diabetes With Loop Use

Participants discussed the psychological impact of living with diabetes both before and since using Loop, with notable improvements expressed in the time spent thinking about diabetes and diabetes-related distress:

Especially for people diagnosed relatively late, whose whole lives have changed, especially with the mental health aspect that diabetes has put a veil over your life, Loop has really helped to stop diabetes being a nuisance and instead it is managed.

24 years, female; 17 years DM; 39 months of Loop use

Participants reported burnout as a result of day-to-day demands, and despite the beneficial impact of Loop on psychological well-being expressed by them, they noted that starting to use the system and the initial setup required a significant investment of time and energy:

I felt burnout in managing my diabetes, spending all my time managing diabetes or filling out insurance forms for my diabetes, it was a real mental challenge to think about and set up a new system, a lot of mental energy.

29 years, female; 27 years DM; 7 months of Loop use

Significant financial investments, both initial and ongoing, were reported by Loop users. They required both the component technology (an insulin pump and a CGM) and appropriate devices on which to set up and use the app—an iPhone with iOS 12.4 or newer operating system and a Mac computer—as well as a communicating device (RileyLink, OrangeLink, or EmaLink) and an Apple developers’ license [25]. Access to and cost of this hardware were the most commonly perceived barriers to Loop use in this cohort:

I would recommend everyone to try it. It is quite a bit of work getting it setup and getting it ready but is pretty minimal effort for upkeep. The access to the devices is the one thing that makes it difficult (especially coverage for it). The peace of mind makes it worth it because it makes so much of a difference.

24 years, female; 14.5 years DM; 5 months of Loop use

Consequently, the use of a system such as Loop comprising multiple devices requires users to ensure that all necessary components are carried around with them and have sufficient battery charge. The devices must be in constant communication with each other to effectively use the app. Some participants reported these day-to-day aspects of Loop use to be challenging at times:

Using Loop there are more things to have to worry about, more tech to charge and make sure you have all the pieces with you when you go places, just more stuff to remember.

31 years, female; 21 years DM; 12 months of Loop use

#### Quantification of Risk

Because Loop is unregulated and therefore unsupported, there may be perceptions of risk. When asked about this, none of the participants considered that using Loop was any more of a risk than an alternate option in diabetes management. Indeed, most participants deemed it to be of much lower risk:

Yes definitely, I am more concerned for the people who don’t use Loop than those who do. It is safer to have a computer system shutting off your insulin and stopping you from going low. It is more trustworthy and makes more rational decisions compared to a person; it shuts off those irrational and emotive decisions so yes, I think it is safer.

30 years, male; 15 years DM; 44 months of Loop use

The importance of setting up the system correctly and “knowing your diabetes” in terms of having the correct insulin pump settings before commencing Loop was expressed by most participants:

I think the only real risk is if there is a lack of understanding that is when problems will arise. I think the system would be risky for newly diagnosed people because we don’t leave the doctor’s office after that first appointment knowing everything, we need to know how to make these systems work. It is a stepwise process but if the settings are set up correctly then I don’t think there are any risks.

49 years, female; 37 years DM; 7 months of Loop use

The limitations of individual components (ie, insulin pump or CGM device), rather than the Loop system itself, were identified as a source of issues that arose during Loop use:

Another challenge or a risk I find during the times when there is a sensor change and the Dexcom is in its warm up period, if the blood sugars haven’t been linking for 2 hours and then it starts, Loop tends to over correct and risks dropping my blood sugars low (which has happened more often than not) it can be a bit better if I allow it to autocorrect.

33 years, female; 22 years DM; 18 months of Loop use

Many participants were using older and out-of-warranty pumps (because many newer in-warranty pumps were incompatible with the Loop app), which they identified as a potential risk in itself:

I was worried about using the older pump but I have recently acquired both a backup pump and RileyLink so have more confidence in this. My pump looks really rough and I do worry occasionally about button errors especially in the heat.

29 years, female; 27 years DM; 7 months of Loop use

Participants reported dissatisfaction with the alternate diabetes management options that are currently available, including Commercial AID systems. A participant had used the Medtronic MiniMed 670G system but had struggled with the Enlite sensor, especially with its alarms. Another user was dissatisfied with Tandem Control-IQ as a result of the lack of customizable glucose targets, with the system providing fixed thresholds that some people felt were too high. Many expressed that they did not wish to consider any other options now that they had experienced Loop:

I feel that Loop is the best option there is right now for people with type 1 diabetes, pump companies are not there yet. I like that people with type one diabetes have built these systems and the #wearenotwaiting movement; the principles and practice of these very gifted individuals who have helped so many people with this technology. I am very thankful to them and just wish more people could have access to it.

33 years, female; 18 years DM; 23 months of Loop use

#### Society’s Understanding and Awareness of Loop

Owing to the unregulated nature of Loop, some participants expressed concern in discussing the use of Loop with others, including with health care providers. All participants in this study were seen in the same diabetes clinic, although with multiple different care providers practicing within the clinic. Most participants expressed positive interactions in the health care setting, frequently describing “passive encouragement” to consider and use Loop. A participant explained that because of the lack of support, with the discouragement of Loop by her previous health care team, she had moved to a new provider as she wished to continue using Loop. Another described being discouraged from continuing to use Loop while seeing a different endocrinologist during pregnancy, despite finding it very beneficial. All other participants felt they could discuss Loop with their clinical team without concern and that health care providers were largely keen to learn more about Loop:

Yes, my healthcare team is very supportive. I have had no negative interactions; I was admitted to the medicine unit – they saw my chart and brought the team in and wanted me to talk about looping and everyone thought it was really cool.

27 years, female; 22 years DM; 40 months of Loop use

Most participants felt that their family and friends were supportive of Loop, although several noted that they had reservations at first, before seeing the benefits of the system for themselves:

There was some hesitancy from my family at first because it’s not government approved; you’re tinkering with it yourself. I see DIY looping as the same as playing around with a pump for programming. Everyone is supportive now. I have friends with diabetes that I have started on loop.

24 years, male; 4.5 years DM; 25 months of Loop use

Many participants had recommended or assisted another person with diabetes in starting Loop, but they indicated that the system may not be beneficial for everyone and felt that prior diabetes education and an understanding of technology were crucial:

Yes, I have helped lots of people with looping, but I would tailor that recommendation based on the individual. Only if they have a good understanding of diabetes management and can critically think through how the system is reacting and what is going on, and interpret the data.

33 years, female; 22 years DM; 18 months of Loop use

Social media, most frequently the Looped Facebook group [26], was a key support structure that all participants had used either currently or previously to set up and troubleshoot Loop. Some noted that through this group, they had been partnered with a current Loop user in a mentor role for further support with starting Loop:

Yes, Looped Facebook group is amazing and so responsive. I also use Alberta diabetes group, Loop and learn and an OrangeLink group. I was set up with a mentor in the Looped group when starting Loop also.

48 years, female; 35 years DM; 3 months of Loop use

Users expressed frustration at the lack of industry support for Loop and the fact that it had required people with diabetes and their families to build this system. However, they also expressed concerns relating to future industry involvement with Loop and the potential changes in the system that this may involve:

I do worry with the increasing success the system may be ‘dumbed down’ in the future and restricted flexibility especially if it is undergoing regulatory approvals with bureaucracy and authorities changing the system.

44 years, female; 32 years DM; 20 months of Loop use

## Discussion

### Principal Findings

In this cohort of adults with T1D at a single center, we have highlighted improved glycemic outcomes with Loop use. With this glucose management system, 67% (16/24) of users achieved the clinically recognized TIR target of 70% [27]. In this first described Canadian cohort of Loop users, we have identified high QOL scores with Loop. The Loop users demonstrated superior glycemic outcomes relative to the general population of people with T1D, with 42% (10/24) of them achieving an HbA_1c_ of <7%, in comparison with the reported average of 21% [13]. The users noted that the removal of an emotive decision-making component in diabetes management was an overwhelmingly favorable aspect of Loop and felt that it aided in the achievement of individualized glucose targets. The safety features of Loop were particularly felt to be important by our participants overnight, with associated improved sleep. Reduction in hypoglycemia (frequency and severity), improved overnight glycemic control, and improved sleep have been widely reported for all DIY AID system types [2,8,28].

We have demonstrated a strikingly similar TIR reported to that in a large prospective observational study of 558 residents of the United States, with a mean age of 23 (SD 13) years who had been new Loop users for 6 months [10]. In this large cohort, with a maximum of 7 days Loop experience at baseline, mean TIR at 6 months was 73% (SD 13%), compared with 71% (SD 16%) in the most recent 6 months of Loop use in our local cohort of 24 users. In comparison to the participants in this prospective study, our study participants were relatively experienced Loop users, with a median of 18 (IQR 12-25) months of Loop use. These results suggest that the benefits of Loop can occur early in its implementation and are somewhat durable, a desirable characteristic for a therapeutic intervention in a chronic condition such as T1D.

No adverse safety outcomes related to hypoglycemia because of Loop use were reported in our data; there was an improvement in TBR, with time <3.0 mmol/L and no admissions related to SH. However, 2 episodes of DKA occurred, both of which were associated with underlying infections. In people with T1D, the estimated incidence of DKA is reported to be 4.6 to 8.0 events per 1000 patient years [29]. Lum et al [10] reported no episodes of DKA with 6 months of Loop in 558 individuals (279 years); they reported 51 episodes of SH, with only one of these episodes being attributed to Loop use [10]. This larger prospective study used weekly electronic messages (with an 89% response rate) for data collection to maximize user recall but was dependent on self-reporting for these likely memorable and significant events for a person with diabetes [30]. Our data were reported based on retrospective recall from participants at the time of interview but were verified by reviews of their medical records. Only 1 of the 2 participants in our cohort self-reported the occurrence of an episode of DKA. All participants in our study reported that they perceived Loop to be safe when the correct settings were in place. Interview responses relating to risk in this cohort were similar to those described by Schipp [20], highlighting a conscious weighing of risks against benefits for DIY AID users. With a detailed understanding of risk, including the use of unregulated and potentially out-of-warranty devices, using a DIY system was felt to be the best glucose management option available to them at this moment in time [19]. DIY AID systems were primarily designed for safety, initially targeting the avoidance of hypoglycemia. This concept of risk reduction through AID system use has been discussed by Lewis [24], highlighting the importance of taking the level of risk in AID use into context, with the risk faced by a person with diabetes who is manually dosing insulin representing the most appropriate comparator and not the risk faced by a person without diabetes. The use of AID systems removes a proportion of this total risk and provides an overall net risk reduction for people with T1D [24].

In terms of quantitative QOL outcomes, we found low diabetes impact and high device satisfaction and INSPIRE scores with Loop use for a median of 18 months in our cohort. The scores were very similar to DIDS outcomes of 2 months of Tandem Control-IQ use (Commercial AID) in 1435 people with T1D aged ≥14 years [15], with a median diabetes impact score of 2.7 (2.8 in this cohort) and median device satisfaction score of 9.1 (9.0 in this cohort). The INSPIRE outcomes of this study were also comparable with those reported with 6 months of Tandem Control-IQ use in another cohort of 112 users with a mean of 87 (IQR 77.6-96.5), in comparison with 86 in this cohort [26]. The median TIR achieved with Tandem Control-IQ was similar to that in our cohort, 79.2% (IQR 70.3%-86.2%) with a shorter duration of AID use, but closer to target glycemia at baseline; with a mean HbA_1c_ of 6.9% (SD 0.9%) [15]. These studies of Commercial AID [17,31] were conducted with substantially greater supervision and support, as would be expected in a randomized controlled trial, in comparison with the real-world experiences collected from our Loop users.

We did not see a strong correlation between device perception and satisfaction outcome measures (DIDS and INSPIRE) or glycemic outcomes in this cohort. This may be a result of the small sample size with a narrow spectrum in these outcomes, but our qualitative data highlight a strong benefit of Loop use on QOL. After improved glycemic outcomes, enhanced QOL was the most frequently reported benefit of Loop use in our cohort. This concept comprises a reduction in the psychological impact of living with diabetes including time spent thinking about diabetes, diabetes-related distress, and burnout, in addition to greater flexibility in day-to-day life, notably related to diet and activity. The reduced mental burden of diabetes and less reliance on the accuracy of carbohydrate counting are consistently reported positive outcomes with DIY AID system use [2].

Another common theme identified was the financial resources required for Loop use, which restricted the availability of this beneficial system. We did not collect data relating to income or index of deprivation, but our participants’ educational attainment and occupations indicated higher-than-average socioeconomic status [32]. Access and coverage of insulin pumps across Canada remains unequal, with varying provincial health care funding models in place; insulin pump therapy is more commonly used in areas with reimbursement programs in place [33].

All except 1 Loop user in this cohort used both an insulin pump and a CGM device at the time of deciding to commence Loop. Having access to, but frequently experiencing dissatisfaction with these devices was a contributing factor to the process of behavior change in these users. For effective behavior change to occur, such as the initiation and continuation of Loop, there are key components for the user and their environment according to the capability, opportunity, motivation, behavior model of behavior change. These include capability (both physical and psychological), physical (including financial and material) and social opportunity (considering social and cultural norms) as well as motivation for change [34]. The components of this model are apparent in the lived experiences that we have described. Loop users highlighted the importance of this physical opportunity, with availability and access to technological devices being a potential limiting factor in the initiation of Loop. Most participants found their health care providers to be relatively supportive toward commencing Loop, despite the system being unregulated. This “social opportunity” enabled reassurance for users, this being an acceptable behavior change. This positive interaction is by no means guaranteed, with varied experiences reported with DIY AID use in health care settings [35].

This study had some strengths and weaknesses. The cohort were recruited from a single center with an integrated medical record that would capture admissions to any facility in the province. Objective collection of these data was performed by the health care team, rather than through self-reporting by users themselves, which has been a weakness in most previous reports of DIY AID systems that describe glycemic outcomes [2,8,11,12,28,36-38]. This study did not include a comparator control group. We collected qualitative data in addition to quantitative data, with Loop users being able to compare their own lived experiences, both with and without Loop use. The sample size for the collection of quantitative outcome data was small, limited by the number of Loop users locally. Selection bias, as a result of the inclusion of individuals who chose to use Loop, must be considered in the generalizability of our findings to the wider population of people with T1D. We have only included current Loop users and therefore, have not been able to explore the reasons behind why users may decide to stop using this form of glucose management system. The fear of disapproval of Loop use from a diabetes care provider as well as barriers to acquiring the component devices have been reported as reasons for Loop discontinuation [22], although we cannot estimate whether these are significant factors in our cohort of individuals who had shared their DIY AID use with their health care providers.

### Conclusions

DIY AID use in this local cohort of individuals who have chosen to start and continue to use Loop has been associated with notable improvements in glycemic outcomes and excellent QOL. Through a combination of quantitative data collection and qualitative interview analysis, we have gained a greater understanding of the lived experiences of the Loop users in this cohort, including the common challenges and extensive benefits. What is most striking is the ability for motivated individuals to further increase their success in achieving glycemic targets while simultaneously experiencing reduced burden and distress from diabetes. Although most DIY users who have been studied to date have been those who were already successful in achieving glycemic targets, future studies should focus on the potential benefits of DIY AID for people who have found it difficult to achieve glycemic targets because of this goal being excessively burdensome or beyond their capacity, as a result of limited financial, social, or educational resources. It is hoped that the experience of Loop users described in this cohort, in combination with further broader user experience, may aid many other future users to access and experience the benefits of Loop use.

## Appendix

Multimedia Appendix 1 Interview transcript, coding structure, and validated quality of life surveys (Diabetes Impact and Device Satisfaction and Insulin Dosing Systems: Perceptions Ideas Reflections and Expectations).

## Abbreviations

AID: automated insulin delivery
CGM: continuous glucose monitor
DIDS: Diabetes Impact and Device Satisfaction
DIY: do-it-yourself
DKA: diabetic ketoacidosis
INSPIRE: Insulin Dosing Systems: Perceptions Ideas Reflections and Expectations
QOL: quality of life
SH: severe hypoglycemia
T1D: type 1 diabetes
TBR: time below range
TIR: time in range
