# Supplementary material for: Improved Glycemia and Quality of Life Among Loop Users: Analysis of Real-world Data From a Single Center
Source: JMIR Diabetes. 2022 Oct 24;7(4):e40326. doi: 10.2196/40326 (PMC9641512; doi:10.2196/40326)
Supplement: Multimedia Appendix 1 [file diabetes_v7i4e40326_app1.docx]

**Multimedia Appendix 1**

**Figure S1. Interview guide**

**Edmonton Do-It-Yourself Automated Insulin Delivery (DIY AID) Systems Users**

**Data Collection Tool**

Thank-you for agreeing to take part in this study, the data we will record will remain anonymous and will only be accessible to our study team. You do not have to take part in all parts of this study, if there are any questions you do not wish to answer you do not have to do so.

*Demographic Data*

1. Age
2. Gender
3. Ethnicity
4. Occupation
5. Highest level of educational attainment
6. Duration of Diabetes (date of diagnosis)
7. Duration of DIY AID use (date commenced)

*Commencing DIY AID*

1. Type of AID used
2. Method of insulin delivery prior to commencing DIY
3. What was the main reason for you to commence DIY AID?
4. What support did you get in doing so?
5. What were the main challenges that you had to overcome in commencing DIY AID?
6. Can you remember what your last HbA1c was prior to commencing DIY AID?
7. Would you be happy for the research team to review your glucose data prior to commencing DIY AID? (HbA1c/CGM)
8. Would you be happy for the research team to review your glucose data for the last 6 months with DIY AID use? (HbA1c/CGM)
9. What is your daily insulin requirement currently?
10. Can you remember what this was before commencing DIY AID?

*Safety of DIY AID*

1. Do you feel that DIY AID systems are safe?
2. Have you had any episodes of severe hypoglycemia since commencing DIY AID? If so, when and why did this occur?
3. Since using DIY AID have you had any episodes of DKA? If so, when and why did this occur?
4. Have you required admission to hospital for any other reason since commencing DIY AID?
5. Have you had any episodes of pump failure?

*Barriers to DIY AID Use*

1. What have you found to be the main barriers in using DIY AID?
2. Do you feel that you have you received adequate support from healthcare providers since switching to DIY AID?
3. Are your family and friends aware that you are using DIY AID and if so have they been supportive of this?
4. Do you feel that there are any risks with using DIY AID, if so what are they?

*Benefits of DIY AID Use*

1. What do you feel to be the main benefits to you of DIY AID use?
2. Are you aware of Commercial AID and if so, what do you think are the benefits of DIY AID relative to commercially approved hybrid closed loop systems?
3. Do you engage in any social media platforms relating to DIY AID use? What do you see as the role and the benefits of these platforms?
4. Would you recommend DIY AID to other people with type 1 diabetes and why?

*Other*

1. Is there anything else that you think is important to discuss about the use of these systems or anything else you would like to mention?

Thank-you for answering these questions and taking part in our research. All answers are anonymized and confidential.

**Figure S2. Interview data coding framework**

**Coding categories and subcategories**

**Quality of Life**

- Lifestyle flexibility – diet, exercise
- Sleep
- Autonomy
- Psychological burden of diabetes- time spent thinking about diabetes, distress and burnout.

**Glucose variability**

- TIR and HbA1c
- Hypoglycemia
- Diabetes complications and co-morbidities
- Pregnancy and female health
- Safety features

**Technology**

- Technology access and sourcing hardware- financial costs
- Knowledgeable in technology ‘techy person’
- Technology issues- connection, carrying all components, battery life, set up time

**User concerns and perceived risk**

- Pre-Loop treatment dissatisfaction
- Fear
- System failure
- Use of old pump
- Incorrect settings
- CGM inaccurate readings

**Support mechanisms**

- Industry
- Social media and other internet resources
- Family and friends

**Local factors**

- HCP
- Coverage- Alberta pump program and Sensor coverage

**Figure S3. DIDS questionnaire**

1. **How satisfied are you with your [insulin delivery device]?**

| Very  Unsatisfied |  |  |  |  |  |  |  |  | Very  Satisfied |
| --- | --- | --- | --- | --- | --- | --- | --- | --- | --- |
| 1 | 2 | 3 | 4 | 5 | 6 | 7 | 8 | 9 | 10 |

1. **How much do you trust your [insulin delivery device]?**

| Not at all |  |  |  |  |  |  |  |  | A lot |
| --- | --- | --- | --- | --- | --- | --- | --- | --- | --- |
| 1 | 2 | 3 | 4 | 5 | 6 | 7 | 8 | 9 | 10 |

**Please indicate how much you agree or disagree with each statement based on your experience using your [insulin delivery device].**

| ***My [current insulin delivery device] …*** | Strongly Disagree  1 | 2 | 3 | 4 | 5 | 6 | 7 | 8 | 9 | Strongly Agree  10 |
| --- | --- | --- | --- | --- | --- | --- | --- | --- | --- | --- |
| 3. …is easy to use. | ○ | ○ | ○ | ○ | ○ | ○ | ○ | ○ | ○ | ○ |
| 4. …helps me have good blood glucose control. | ○ | ○ | ○ | ○ | ○ | ○ | ○ | ○ | ○ | ○ |
| 5. …is a hassle to use. | ○ | ○ | ○ | ○ | ○ | ○ | ○ | ○ | ○ | ○ |
| 6. …helps me feel more in control of my diabetes. | ○ | ○ | ○ | ○ | ○ | ○ | ○ | ○ | ○ | ○ |
| 7. …is too complicated. | ○ | ○ | ○ | ○ | ○ | ○ | ○ | ○ | ○ | ○ |

**How often do you…?**

|  | Never  1 | 2 | 3 | 4 | 5 | 6 | 7 | 8 | 9 | Always 10 |
| --- | --- | --- | --- | --- | --- | --- | --- | --- | --- | --- |
| 8. …have a bad night sleep due to diabetes? | ○ | ○ | ○ | ○ | ○ | ○ | ○ | ○ | ○ | ○ |
| 9. …wake up at night to treat a low blood glucose? | ○ | ○ | ○ | ○ | ○ | ○ | ○ | ○ | ○ | ○ |
| 10. …worry about going low? | ○ | ○ | ○ | ○ | ○ | ○ | ○ | ○ | ○ | ○ |
| 11. …miss work, school, chores, or other responsibilities due to diabetes? | ○ | ○ | ○ | ○ | ○ | ○ | ○ | ○ | ○ | ○ |

**Scoring Instructions:**

# Device Satisfaction: Average of items 1 - 7 (#5, #7 are reverse scored)

# Diabetes Impact: Average of items 8 - 11

**Figure S4. INSPIRE questionnaire**

INSPIRE Questionnaire for Adults with Type 1 Diabetes (Post Intervention)

We would like to ask about your thoughts and feelings about your experience using an automated insulin dosing system (**abbreviated AID**), sometimes called a closed loop system, artificial pancreas or bionic pancreas. We would like you to think about living with diabetes and the things that may have been better or worse by using **AID**. **For each of the questions below, please tick (check) the box that best fits your answer. Please answer every question.**

|  |  | Strongly Agree | Agree | Neither  Agree nor Disagree | | Disagree | Strongly Disagree | N/A |
| --- | --- | --- | --- | --- | --- | --- | --- | --- |
| 1 | I was more hopeful about my future when using automated insulin dosing (AID). | □ | □ | □ | | □ | □ |  |
| 2 | I worried less about diabetes with AID. | □ | □ | □ | | □ | □ |  |
| 3 | AID reduced my family’s concerns about my diabetes. | □ | □ | □ | | □ | □ |  |
| 4 | AID made it easier for me do the things that I wanted to do without diabetes getting in the way. | □ | □ | □ | | □ | □ |  |
| 5 | AID decreased how often I had low glucose levels. | □ | □ | □ | | □ | □ |  |
| 6 | AID decreased how often I had high glucose levels. | □ | □ | □ | | □ | □ |  |
| 7 | AID helped me stay in my target range more often. | □ | □ | □ | | □ | □ |  |
| 8 | AID improved my A1c to target level. | □ | □ | □ | | □ | □ |  |
| 9 | AID made it easier to eat when I wanted to. | □ | □ | □ | | □ | □ |  |
| 10 | AID made it easier to exercise when I wanted to. | □ | □ | □ | | □ | □ |  |
|  |  | Strongly Agree | Agree | Neither  Agree nor Disagree | Disagree | | Strongly Disagree | N/A |
| 11 | AID made managing diabetes easier when I was at work or school. | □ | □ | □ | □ | | □ |  |
| 12 | AID made managing diabetes easier when it came to my social life/being with friends. | □ | □ | □ | □ | | □ |  |
| 13 | AID helped me manage sick days. | □ | □ | □ | □ | | □ |  |
| 14 | AID helped me sleep better. | □ | □ | □ | □ | | □ |  |
| 15 | AID helped me have fewer hypos during the night | □ | □ | □ | □ | | □ |  |
| 16 | AID improved my overall quality of life. | □ | □ | □ | □ | | □ |  |
| 17 | AID improved my family’s overall quality of life. | □ | □ | □ | □ | | □ |  |
|  |  | Strongly Agree | Agree | Neither Agree nor  Disagree | Disagree | | Strongly Disagree | N/A |
| 18 | AID made managing diabetes easier when driving (for those who drive) or when travelling | □ | □ | □ | □ | | □ |  |
| 19 | AID helped me manage diabetes when it came to my sex life | □ | □ | □ | □ | | □ |  |
| 20 | AID helped me manage my diabetes when I drank alcohol | □ | □ | □ | □ | | □ |  |
| 21 | AID helped me when I was pregnant | □ | □ | □ | □ | | □ |  |
| 22 | AID reduced my risk of long-term complications. | □ | □ | □ | □ | | □ |  |

Thank you for taking part, your answers are very important to us.
